# A study of animal welfare in 11 species across 16 Zoos using M-AWAG and its correlations with environmental variables

**DOI:** 10.3389/fvets.2026.1814857

**Published:** 2026-05-19

**Authors:** Hyejin Kang

**Affiliations:** Department of Horse/Companion and Wild Animal Science, Kyungpook National University, Daegu, Republic of Korea

**Keywords:** animal welfare assessment, animal welfare assessment grid, microenvironment, South Korea, zoo animal welfare, zoo animals, zoo welfare inspection

## Abstract

In 2023, there were 114 registered zoos in Korea, more than half of which are small indoor zoos. The welfare conditions of these indoor zoos have become a social issue. The amendment to the Zoo and Aquarium Act in 2023 introduced a licensing system based on the UK’s zoo licensing framework, requiring zoos to meet specific criteria in order to obtain an operating license. This study conducts welfare assessments of zoo animals, allowing for the evaluation of the welfare status of each species and the welfare levels associated with the zoo environment. This study assessed animal welfare and microenvironmental conditions in South Korean zoos using a pilot inspector system integrating the Modified Animal Welfare Assessment Grid (M-AWAG) with environmental evaluations. Sixteen zoos were selected from 109 registered facilities, categorized into four groups based on accreditation status and size. Eleven commonly housed species were evaluated to maximize species diversity. Three trained inspectors—a wildlife veterinarian, a zookeeper, and an animal welfare researcher—conducted welfare assessments across four categories: physical, psychological, environmental, and procedural. Environmental variables measured included temperature, humidity, sound levels, illuminance, and odor concentration. The inter-rater reliability among inspectors was 0.942 with high objectivity. Results showed environmental (scores ranging from 4.58 to 6.95), psychological (3.38–4.42), and procedural (3.54–4.78) welfare scores were consistently poorer than physical scores (1.63–2.51) across all species, indicating significant areas for improvement. Analysis of environmental variables showed that illuminance, temperature, and humidity varied significantly among zoo groups Higher sound levels were associated with higher food and drink intake scores (r = 0.28, *p* < 0.001). High illuminance was negatively correlated with food and drink intake scores (r = −0.24, *p* = 0.007). Relative humidity was negatively correlated with food and drink intake (r = −0.17, *p* = 0.046) and activity level (r = −0.18, *p* = 0.042). The results of this study, conducted as the first pilot project of its kind in Korea, demonstrate that microenvironmental factors can influence animal welfare and highlight the importance of individualized welfare assessments tailored to the ecological and behavioral needs of each species. The standardized welfare assessment protocol developed in this study can provide an empirical basis for evaluating and improving the welfare of animals of various species in domestic zoos, and easily measurable environmental-based assessments—such as temperature, humidity, light, and sound—provide essential data for improving actual animal welfare.

## 1 Introduction

The environment in which captive animals are maintained has a direct and significant impact on their welfare—including health and behavior—unlike that of their wild counterparts (1, 2). Husbandry environments that disregard the natural habits of animals often inhibit the expression of their instinctive and species-typical behaviors, potentially leading to behavioral deficiencies when these conditions fail to align with the animals’ ecological and natural history (2–5). As a result, the behavioral and physiological needs of captive animals may not be adequately met, leading to negative effects on their overall welfare. This issue is particularly pronounced in zoos, where animals are confined under conditions vastly different from their natural habitats. Such artificial environments can significantly alter their behavior and physiological responses (4–7). For instance, temperature and humidity are critical for maintaining physiological balance; improper management of these factors can increase stress levels (1, 4, 8). Additionally, exposure to unfamiliar odors and excessive sound can disrupt psychological stability, heightening anxiety and triggering abnormal behaviors (9–13).

Since the formal inception of animal welfare research, extensive studies have focused on farm animals, examining various environmental factors that influence productivity, behavior, and health (14–18). Research in farm animal welfare evaluates not only the physical environment—such as flooring materials, bedding, and outdoor access areas—but also microenvironmental elements like temperature, humidity, odors, and airborne bacterial concentrations (19–22). This progress in farm animal research is largely attributable to the economic importance of farm animals, where welfare conditions are directly linked to productivity (23–25). Consequently, improving farm animal welfare is recognized as offering mutual benefits for both economic gains and animal well-being. In contrast, research specifically examining microenvironmental factors within zoos is lacking compared to the substantial body of work on farm animal welfare (8–10). Although considerable research exists on general zoo animal welfare, the impact of microenvironmental elements on the well-being of zoo animals has been largely overlooked (26–28).

Understanding and optimizing microenvironmental factors such as temperature, humidity, sound, and light is crucial for addressing the physical and psychological needs of captive animals (2, 5, 29). Proper environmental management can enhance animal welfare, support conservation efforts, and improve both educational value and visitor satisfaction (6, 7, 30). While there have been numerous systematic studies on the microenvironments of farm animals, research on the microenvironments of zoo animals is limited (21, 31). This research gap can be attributed to several factors. First, most zoos house a small number of animals across a wide variety of species, each with unique characteristics and environmental needs, making it challenging to apply a standardized assessment framework across all animals (26, 27, 32). This diversity and limited sample size make it difficult to generalize results that are tailored to specific species (6, 32, 33). Additionally, assessing zoo animals individually requires substantial time and resources for customized assessments, which increases the cost of research (6, 26, 31). As a result, existing studies often fail to adopt a customized approach, leading to generalized conclusions that overlook species-specific characteristics (26, 31, 34). The lack of species-specific data hinders the development of effective environmental management practices, potentially compromising animal welfare.

Second, the primary operational objectives of many Korean zoos focus on attracting visitors and providing entertainment, which limits resources allocated to animal welfare research (30, 31, 35). This commercial emphasis often leads to welfare improvements being deprioritized in favor of revenue-generating activities (30, 35). Consequently, there is a shortage of funding for systematic animal welfare studies, including environmental research. This scarcity makes it challenging to conduct thorough investigations and leads to the use of welfare assessment tools and methods that have not been sufficiently validated (34). The inadequate validation and improper application of these tools further undermine the reliability of existing research and impede evidence-based improvements. Third, unlike farm animal welfare evaluations that often utilize standardized assessment tools, zoo animal welfare assessments employ a variety of methods and approaches (26, 33, 34). This methodological diversity aims to reflect individual species’ characteristics; however, it reduces the reliability of research findings and complicates comparisons between studies (6, 7, 26). As a result, these factors have slowed environmental research in domestic zoos. Without consistent data, policymakers and zoo managers lack the necessary information to make informed decisions about environmental modifications that could improve animal welfare. Therefore, there is an urgent need for systematic research to elucidate the relationship between microenvironmental factors and the welfare of zoo animals in South Korea.

From 2016 to the present, South Korea’s zoo regulations have undergone changes to address animal welfare issues. As of 2023, there are 114 registered zoos (36), with more than half being indoor zoos focused on animal interaction programs (31, 35). The welfare conditions in these indoor zoos have become a social concern, drawing significant criticism from animal rights groups (37, 38). In response, the Korean government enacted the “Act on the Management of Zoos and Aquariums” in 2016 (39), implementing a zoo registration system. However, this system had limitations, allowing zoo operations merely upon registration without significantly improving animal welfare (40). The law required facilities housing more than 10 species or more than 50 individual wild animals to register as zoos, excluding smaller facilities from registration. This exclusion created a legal blind spot, causing serious issues in animal welfare and safety management. Consequently, many small wild animal cafés and private zoos operated without regulation, exploiting loopholes to avoid welfare regulations while functioning as establishments selling food and beverages.

By 2021, concerns over animal welfare intensified as investigations revealed about 150 registered zoos and potentially up to 300 similar facilities (41). During this period, the zoo law did not specify suitable habitats or minimum area standards for animals (40). Consequently, wild animals were often confined in small spaces, causing significant stress and impacting their health and survival (1, 2, 5). These animals exhibited abnormal behaviors such as colliding with each other or displaying repetitive stereotypic behaviors. Although environmental enrichment is essential to alleviate boredom and reduce stereotypies, many indoor zoos lacked facilities for such enrichment (7, 26, 35). Additionally, many indoor zoos operated feeding experience programs that forced animals into unnatural behaviors inconsistent with their innate habits (31, 35, 38). These programs disregarded ecological habits and overly exposed animals to humans, leading some to become habituated to waiting for food from visitors.

In response, on September 11, 2020, a legislative bill was proposed to restrict and manage the import, distribution, and contact with wild animals (42, 43). This bill included transitioning the zoo registration system to a permit system, restricting operator qualifications, providing appropriate habitats, and managing zoonotic diseases. It also established regulations for small zoos, set habitat environmental standards, limited interactive events, regulated animal transfers upon closure, and introduced an inspector system. Building on this initiative, significant amendments (6) were made in 2023 to enhance animal welfare standards (44). One major change was prohibiting experience activities that induce stress in animals, effectively abolishing practices that force unnatural behaviors (45, 46). Despite these reforms, recent studies have shown that wild animal cafés continue to operate as before the amendments, highlighting the limitations of legislative measures without strong enforcement and oversight (35).

The 2023 amendment also introduced a permit system based on the UK’s zoo licensing framework, requiring zoos to meet certain standards to obtain operating permits (35, 38). While this system effectively prevented non-compliant zoos from operating, it relied heavily on subjective inspections (26, 34). Additionally, a shortage of inspectors with sufficient knowledge and experience limited meticulous evaluation of individual animal welfare (31, 35). To address these limitations, an inspector system responsible for regular evaluations and random inspections of animal welfare was introduced^6^. This newly implemented system offers a potential solution by providing consistent welfare assessments across all facilities, including previously unregulated small animal cafés (37, 38, 47). Inspectors with specialized training and expertise can identify non-compliance, enforce regulations, and ensure adherence to updated welfare standards. By systematically monitoring both large, accredited zoos and smaller uncertified facilities, the inspector system aims to close legal gaps and significantly improve zoo animal welfare in South Korea.

This study was conducted from July to August 2020, before the full implementation of the current zoo regulatory framework in the Republic of Korea. In particular, the revised Act on the Management of Zoos and Aquariums, which introduced the permit-based system and the formal zoo inspector system, entered into force on December 14, 2023; therefore, the present study should be understood as a pre-implementation pilot assessment rather than an evaluation of the effectiveness of the current inspection framework. Inspectors are experts whose qualifications are stipulated by Presidential Decree, consisting of individuals who have obtained veterinary licenses and worked in zoos for a certain period or those with specialized knowledge in animal welfare. In the 2020 study by Ma et al., three experts were appointed as pilot inspectors to visit 16 zoos for a month (31). Our study was also conducted during this period using the same methodology. While Ma’s study demonstrated statistically significant differences and high inter-rater consistency (IRR = 0.942) in the animal welfare levels of domestic zoos divided into four groups according to accreditation status, size, species and number of individuals, it did not comprehensively cover the welfare levels of all 11 species and lacked an in-depth analysis of the microenvironment (31, 35).

Against this background, our study is the first in South Korea to systematically analyze the impact of the microenvironment in zoos on animal welfare. It aims to evaluate animal welfare levels according to species and taxonomic groups and quantitatively analyze the correlation between animal-based Modified Animal Welfare Assessment Grid (M-AWAG) scores and microenvironmental variables. Through this, we seek to identify how environmental factors related to animal welfare affect the health and behavior of animals and provide empirical evidence for the effective operation of the inspector system. Furthermore, by providing data that objectively assess animal welfare in zoo environments, this study will lay important groundwork for designing environments that can maximize welfare levels and encourage animals’ natural behaviors. Such research is expected to contribute to improving the overall animal welfare levels of zoos in South Korea by preparing customized welfare management plans based on evaluations according to species and taxonomic groups.

This study aimed to systematically examine the relationship between microenvironmental factors and animal welfare outcomes across multiple zoos in South Korea, using the M-AWAG framework in conjunction with a pilot inspector system.

## 2 Materials and methods

### 2.1 Researchers

This study was conducted as a pilot program for three animal welfare assessors prior to the implementation of the Animal Welfare Assessor Program. The inspectors included a wildlife veterinarian with 22 years of experience, a mammal, bird, and reptile keeper with 35 years of experience, and an animal welfare researcher with 14 years of experience in conducting animal welfare assessments. They underwent standardized training, which involved familiarizing themselves with the Animal Welfare Assessment Grid (AWAG) protocol through relevant literature and completing several practice sessions using the M-AWAG at various zoos in Korea. During this training process, the inspectors systematically learned the specific definitions and evaluation methods for each item of the AWAG and M-AWAG, and repeatedly compared and adjusted their assessment results in identical scenarios to enhance consistency among evaluators. These inspectors visited 16 zoos across Korea to perform welfare evaluations. In most zoos, they reviewed management records prior to conducting assessments and collaborated with zookeepers to visit enclosures and seek opinions and advice on the animals’ status. The evaluations were conducted in a blinded manner using personal mobile devices, and discussions among inspectors were limited to necessary cases, such as verifying the number of injured animals that were difficult to observe. Assessments in large zoos totaled 68 h, while those in small zoos amounted to 45 h. Inter-observer reliability was evaluated.

### 2.2 Welfare-assessed zoos

The study adhered to ethical standards and maintained appropriate hygiene management throughout its execution. During the summer of 2020 (July–August), welfare assessments were conducted on 11 species across 16 zoos in Korea. Prior to visiting the zoos, the Ministry of Environment requested each zoo’s local government to notify and seek cooperation from the respective zoos. Out of a total of 109 registered zoos, two were excluded due to closures, leaving 107 zoos. These were categorized into four groups (A, B, C, D) based on accreditation status, size, species and number of individuals the number of animals, and operational systems to facilitate the assessment and comparison of animal welfare levels according to each group’s characteristics and scale (Table 1).

**Table 1:** The standard of the grade for 16 zoos assessed in Korea.

Zoo grade	Standard of the zoo grade	No. of zoos	Assessed zoos	Ratio (%)
A	AZA-accredited zoos^*^	2	2	100%
B	KAZA certified zoos^**^	11	4	36%
C	Large uncertified zoos, ≥ 50 species, ≥1,000 animals, ≥3,000 m^2^	38	4	10%
D	Small uncertified zoos, < 50 species, <1,000 animals, <3,000 m^2^	56	6	10%
	Total	107	16	15%

This table illustrates the grading standards for 16 zoos assessed in South Korea, categorized into four levels: A, B, C, and D. The A grade includes AZA-accredited zoos, with 2 out of 2 zoos assessed, showing a 100% evaluation rate. The B grade consists of KAZA-certified zoos, where 4 out of 11 zoos were evaluated, representing a 36% evaluation rate. Grade A, which is internationally recognized, ensures higher welfare standards compared to KAZA, which holds domestic recognition. The C grade encompasses large, uncertified zoos that have more than 50 species, 1,000 animals, and over 3,000 m^2^ of space. Out of 38 zoos in this category, only 4 (10%) were assessed. The D grade represents small, uncertified zoos with fewer than 50 species, less than 1,000 animals, and under 3,000 m^2^ of space. In this group, 6 out of 56 zoos (10%) were evaluated. Overall, only about 15% of all zoos in South Korea were assessed, providing an insight into the welfare evaluation status and certification levels of zoos in the country (^*^The AZA (Association of Zoos and Aquariums) recognized globally for high animal welfare standards; ^**^ the KAZA (Korea Association of Zoos and Aquariums) maintain a certain level of welfare standards; AZA accredited zoos are internationally recognized, ensures higher welfare standards compared to KAZA, which holds domestic recognition).

The A grade zoos accredited by the Association of Zoos and Aquariums (AZA) (48), recognized globally for high animal welfare standards, with 2 out of 2 zoos assessed, showing a 100% evaluation rate. Group B consisted of zoos accredited by the Korea Association of Zoos and Aquariums (KAZA) (49), which maintain a certain level of welfare standards, where 4 out of 11 zoos were evaluated, representing a 36% evaluation rate. Grade A, which is internationally recognized, ensures higher welfare standards compared to KAZA, which holds domestic recognition. The C grade encompasses large, uncertified zoos that have more than 50 species, 1,000 animals, or over 3,000 m^2^ of space. Out of 38 zoos in this category 4 (10%) zoos were assessed. The D grade represents small, uncertified zoos with fewer than 50 species, less than 1,000 animals, and under 3,000 m^2^ of space. In this group, 6 out of 56 zoos (10%) were evaluated. Overall, about 15% of all zoos in South Korea were assessed, providing an insight into the welfare evaluation status and certification levels of zoos in the country.

### 2.3 Target animals

Based on the animal registration data from the Korean Ministry of Environment, we reviewed the animal lists classified into 11 groups and selected 11 representative species commonly housed within each group to maximize the diversity of species assessed. Consequently, welfare evaluations were conducted on 11 species totaling 153 individual animals across 16 zoos. The target species included black-tailed prairie dog (*Cynomys ludovicianus*), European rabbit (*Oryctolagus cuniculus*), Japanese macaque (*Macaca fuscata*), raccoon (*Procyon lotor*), fennec fox (*Vulpes zerda*), meerkat (*Suricata suricatta*), Siberian tiger (*Panthera tigris altaica*), blue-and-yellow macaw and red-and-green macaw (*Ara ararauna* and *Ara chloropterus;* The two species originate from different geographical distribution areas and may exhibit slight ecological differences in the wild; however, due to similar husbandry conditions, they are often kept under the same environmental conditions in zoos.), cockatiel (*Nymphicus hollandicus*), Burmese python (*Python bivittatus*), and African spurred tortoise (*Centrochelys sulcata*). Animals that were sick or injured and thus not on exhibit were not included in the assessment.

The primary observational unit in this study was the individual animal. However, because animals were housed within shared enclosures and zoological institutions, the data had a hierarchical structure, with individuals nested within enclosures and enclosures nested within zoos. Animal-based indicators were recorded for each individual, whereas enclosure-based environmental measures were recorded at the enclosure level and linked to all individuals housed in that enclosure.

### 2.4 Modified animal welfare assessment grid (M-AWAG)

This study utilized the Animal Welfare Assessment Grid (AWAG) proposed by Justice et al. (32) for assessing the welfare of zoo animals. AWAG is designed to evaluate individual animals by categorizing welfare into four parameters: physical, psychological, environmental, and procedural, each quantified on a 10-point scale (see the modified AWAG in Supplementary Tables 1–4). To better suit the Korean context, enhance practicality, and improve efficiency, the existing AWAG was adapted into a Modified Animal Welfare Assessment Grid (M-AWAG) framework. AWAG is structured on a 10-point Likert scale, ranging from 1 to 10. The criteria are separated by very small differences, which makes the evaluation process more complex and time-consuming, particularly for domestic use. The M-AWAG employs a 6-point Likert scale for assessment, utilizing the following scores: 1; 3 (combining scores 2 and 3); 5 (combining scores 4 and 5); 7 (combining scores 6 and 7); 9 (combining scores 8 and 9); and 10 (31). In this scale, lower scores indicate better conditions, while higher scores denote poorer conditions. For instance, a score of 1 represents the best possible condition, whereas a score of 10 signifies the worst. This modification aligns with the tools used by Ma et al. in 2020, ensuring consistency and comparability with previous research (31). The evaluation criteria for M-AWAG were pre-formatted into Google Forms, comprising 35 survey questions. Three inspectors independently recorded scores for each of the 153 animals across these questions using Google Forms (Google, Mountain View, CA), which automatically processed the statistical data. The assessment covered the following elements:

- Physical parameters: physical parameters included general condition (weight, body condition score, coat/feather condition), clinical assessment (injuries, alopecia, vomiting, other clinical symptoms), fecal consistency (not assessed in birds), activity level (activity and mobility), and food & drink intake (not eating/drinking or reported hunger/thirst).
- Psychological parameters: psychological parameters assessed abnormal behaviors (automutilation, regurgitation, stereotypies), responses to catching events, social hierarchy (upsets/disputes, aggression/bullying), enrichment (provision and use), aversion to normal events, and training (not assessed in birds).
- Environmental parameters: environmental parameters covered housing (adequate space, lighting, ventilation, accessible shelters, materials used, temperature, drainage, humidity, UV where necessary, low sound levels), group size (alignment with natural group size, appropriate stocking density, suitable group structure), furnishing/enclosure design (branches, plants, hiding places, shelters, nest boxes, items facilitating natural behaviors), nutrition (diet and forage suited to species-specific needs), and access and contingent events (animal movement, enclosure changes, building works, visitor interactions). Considering the suboptimal animal housing conditions domestically, environmental parameters were assessed in greater detail, incorporating specific criteria to ensure a comprehensive evaluation.
- Procedural parameters: procedural parameters included restraint (training/habituation to restraint), sedation (stress from sedation, recovery), time restrained before/during procedures, veterinary procedures, changes in daily routine (starvation hours, housing changes, solitary housing), and visitor score (visitor numbers, sound levels, adverse interactions).

Upon completion of the assessments, the M-AWAG scores recorded in Google Forms were averaged for each animal to derive the final M-AWAG score.

### 2.5 Environment variable measurement tools

In addition to the M-AWAG, various measurement tools were employed to comprehensively evaluate the breeding environments impacting animal welfare. All measurements were conducted in triplicate to ensure accuracy and reliability.

Temperature and relative humidity were measured at three distinct points within each enclosure where animals typically rested, using a hygrothermograph (SK-110TRH, SATO, Tokyo, Japan). Bacterial counts were assessed for Japanese macaques, fennec foxes, and black-tailed prairie dog s—among the 11 common species of carnivores, omnivores, and herbivores evaluated across the 16 zoos. Three types of microorganisms were tested (CFU/100cm^2^): *Escherichia coli* counts, coliform counts, and aerobic counts. Samples were collected from two specific locations within each exhibit: the drinking water area and the resting area. Each sample was taken in triplicate (10×10 cm) using a 3 M Pipette Swab (Saline), stored in a cooler, and tested within 72 h. For *E. coli* and coliform colonies, 1 mL of the sample was inoculated onto 3 M^™^ Petrifilm^™^ *E. coli* Count Plates and incubated at 36 °C for 24 h. For general bacteria, 1 mL of the sample was inoculated onto 3 M^™^ Petrifilm Aerobic Count Plates and incubated at 36 °C for 48 h. Odor levels (ppm) were measured using an odor detection device (OMX-ADM, Shinyei, Japan) due to its simplicity and rapid measurement capabilities. Gas samples were collected at the resting sites within animal enclosures. Sound levels (dB) were assessed using a Soundmeter (TES-53S, Taiwan) at the resting sites, and illuminance levels (lux) were measured with a light meter (FLUKE-941, United States) based on the brightest areas within each enclosure.

### 2.6 Statistical analysis

#### 2.6.1 Descriptive statistics

Descriptive statistics were conducted using Excel to summarize the 23 M-AWAG scores for 11 species across 16 zoos, as measured by three inspectors. Frequency tables and graphs were created to visually represent the mean and standard deviation of each variable, facilitating an overview of the general trends and distributions within the data.

#### 2.6.2 Inter-rater reliability of modified AWAG

This study evaluated the inter-rater reliability (IRR) of AWAG scores among the veterinarians, zookeepers, and researchers. Fleiss’ kappa was measured to evaluate the IRR of AWAG scores among all three inspectors. A linear-weighted Cohen’s kappa was used to measure the IRRs of each pair of inspectors. If IRR is 0.9 or more, it can be considered that the reliability is high. These assessments were performed using the “Irr” package in R (R Foundation, Vienna, Austria) (50).

#### 2.6.3 Statistical differences and correlation analysis

Data analysis was performed using R Studio version 4.2.1 through a multi-step process. Initially, the dependent variables were defined as physical, psychological, procedural, and environmental factors. The 16 zoos were categorized into four groups (A, B, C, D), and differences between these groups were compared for each animal species. First, descriptive statistics were conducted to determine the mean and standard deviation for each group, enabling the identification of general trends and facilitating visual comparisons between groups. Second, the Kruskal-Wallis test was employed to assess differences among the four zoo groups for the 11 animal species. This non-parametric test was chosen due to the small sample size and potential non-normal distribution of the data. When significant differences were identified through the Kruskal-Wallis test, Dunn’s test was performed as a post-hoc analysis to pinpoint specific group comparisons contributing to the overall significance.

Third, the correlation between microbial counts and environmental variables was examined using Spearman’s rank correlation analysis. The Spearman correlation coefficient (r) was interpreted as follows: 0.0 < r ≤ 0.2 indicated a very weak correlation, 0.2 < r ≤ 0.4 indicated a weak correlation, 0.4 < r ≤ 0.6 indicated a moderate correlation, 0.6 < r ≤ 0.8 indicated a strong correlation, and 0.8 < r ≤ 1.0 indicated a very strong correlation. This analysis aimed to identify relationships between microbial presence and various environmental conditions, thereby evaluating the impact of environmental variables on animal welfare. Finally, results were visually represented using radar charts in R Studio to provide a comprehensive view of the correlations between different variables. Statistical significance was determined at *p* < 0.05.

## 3 Results

The modified AWAG showed an IRR of 0.942 for all three inspectors’ evaluations. The score for each inspector was high (> 0.95) (Table 2). In this study, the welfare status of 11 animal species across 16 zoos in South Korea was assessed using the Modified Animal Welfare Assessment Grid (M-AWAG). The target zoos were classified into four groups based on their accreditation and size: AZA-certified zoos (Group A), KAZA-certified zoos (Group B), large non-certified zoos (Group C), and small non-certified zoos (Group D) (Table 1).

**Table 2:** Inter-rater reliability (IRR) between each pair of inspectors and all three inspectors.

Inspectors	Veterinarian & zookeeper	Zookeeper & researcher	Researcher & veterinarian	All inspectors
Inter-rater reliability	0.963	0.965	0.971	0.942

The Fleiss’ Kappa was used to evaluate inter-rater reliability of AWAG scores among all three inspectors. The linearly weighted Cohen’s Kappa was used to measure inter-rater reliabilities of each pair of inspectors.

**Figure 1:**
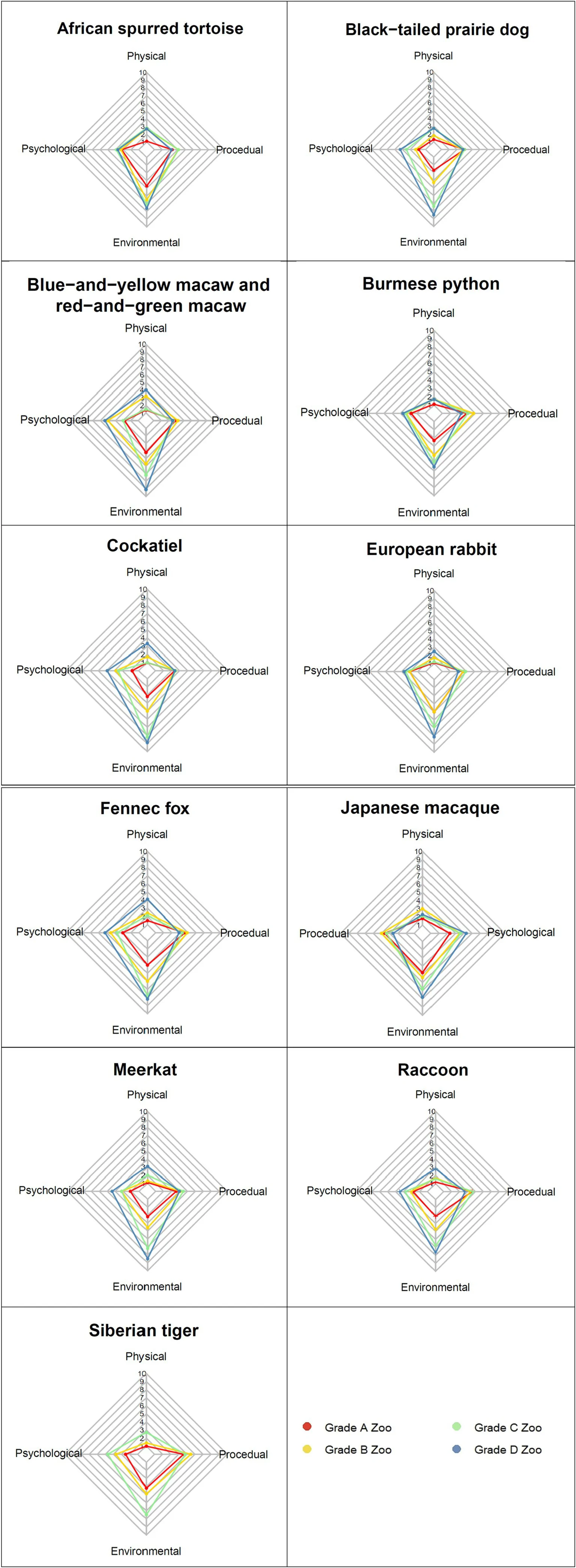
The mean scores across the four categories of the Modified Animal Welfare Assessment Grid (M-AWAG) were calculated for 11 species distributed among zoo classes A, B, C, and D. The radar chart presents the Modified Animal Welfare Assessment Grid (M-AWAG) scores across four categories—physical, psychological, procedural, and environmental—for 11 species evaluated in 16 South Korean zoos. The chart distinguishes between the four zoo groups with specific color coding: Group A (AZA-accredited zoos) is in red, Group B (KAZA-certified zoos) in yellow, Group C (large uncertified zoos) in green, and Group D (small uncertified zoos) in blue. Each point on the radar chart represents the average score for a specific welfare category, with lower scores indicating better welfare conditions and higher scores denoting poorer conditions. The radar chart’s size variation for each group visually conveys the differences in welfare conditions, with larger chart areas representing poorer overall welfare. Specifically, the scores demonstrate that environmental management and procedural factors are areas needing improvement across all groups, but these issues are most pronounced in Group D. The chart provides a clear visual indication of how welfare conditions vary significantly based on zoo accreditation and management quality, emphasizing the importance of accreditation and systematic management in maintaining high welfare standards.

Figure 1 presents a radar chart illustrating the welfare status of the 11 species, where lower scores indicate better welfare conditions. Group A zoos are depicted in red, Group B in yellow, Group C in green, and Group D in blue. Upon comparing the average M-AWAG scores across the four categories—physical, psychological, procedural, and environmental—it was observed that environmental scores (4.58–6.95), psychological scores (3.38–4.42), and procedural scores (3.54–4.78) were consistently higher than physical scores (1.63–2.51) across all species. This pattern was consistent for every species evaluated. Additionally, M-AWAG scores showed a progressive increase from Group A to Group D, indicating that zoos in Group D generally exhibited poorer welfare conditions compared to those in Groups A, B, and C. Overall, Groups A and B recorded lower scores across all evaluation categories, reflecting better welfare conditions. However, it was noted that not all evaluation categories were uniformly excellent across these groups. Specifically, certain species within Groups A and B demonstrated areas needing improvement. For instance, Cockatiels and blue-and-yellow macaws and red-and-green macaws in Groups A and B scored relatively higher in the procedural category, suggesting deficiencies in procedural management, such as regulations and management protocols for these animals. Similarly, Meerkats in Groups A and B scored somewhat higher in the environmental category, indicating a need for improved habitat management. Conversely, some positive outcomes were observed even in Group D zoos. Japanese Macaques and Pythons in Group D recorded physical scores comparable to those in Group A zoos, suggesting relatively good physical management despite the overall poorer welfare status of Group D zoos. Furthermore, in procedural scores, Group D zoos maintained relatively lower scores similar to Groups A and B, indicating that procedural management was not significantly different across these groups.

The M-AWAG score differences among Groups A-D for each animal species were analyzed using the Kruskal-Wallis test, as presented in Table 3 and Supplementary Table 5. The results indicated no statistically significant differences among the groups for Japanese macaques, Siberian tigers, fennec foxes, and pythons. However, significant differences were observed for black-tailed prairie dogs, meerkats, European rabbits, African spurred tortoise, blue-and-yellow macaw and red-and-green macaws, raccoons, and cockatiels (*p* < 0.05). For cases where significant differences were identified through the Kruskal-Wallis test, a *post hoc* Dunn’s test was conducted to determine specific inter-group differences. *Post hoc* analyses revealed that: For black-tailed prairie dogs, Group D recorded significantly higher scores in both psychological (*p =* 0.044) and environmental categories (*p =* 0.040) compared to Groups A and B, indicating poorer welfare conditions. For Meerkats, Group D scored significantly higher in physical (*p =* 0.008) and environmental categories (*p =* 0.009) compared to Groups A and B, and higher in the psychological category compared to Group A (*p =* 0.010). For European rabbits, Group D scored significantly higher in the physical category compared to Group C (*p =* 0.021) and in the environmental category compared to Group B (*p =* 0.007), reflecting inadequate welfare conditions.

**Table 3:** The differences in physical, psychological, procedure, environmental scores according to zoos.

Species	AWAG	A	B	C	D	*P*	Post hoc
Black-tailed prairie dogs	Physical	1.30 ± 0.28	1.80 ± 0.57	2.70 ± 0.46	2.76 ± 1.23	0.109	A = B=C=D
	Psychological	1.95 ± 0.50	3.53 ± 1.21	3.53 ± 1.21	4.30 ± 0.71	0.044*	D > A, D > B
	Procedure	4.00 ± 0.00	3.80 ± 0.28	4.03 ± 0.59	3.84 ± 1.17	0.723	A = B=C=D
	Environmental	2.65 ± 0.49	4.30 ± 2.26	7.37 ± 0.35	8.42 ± 1.28	0.040*	D > A, D > B
Meerkats	Physical	1.05 ± 0.70	1.28 ± 0.25	2.00 ± 0.75	3.10 ± 1.00	0.008	D > A, D > B
	Psychological	2.05 ± 0.49	3.18 ± 0.67	3.18 ± 0.59	4.43 ± 0.78	0.010*	D > A
	Procedure	3.60 ± 0.00	4.13 ± 0.19	4.45 ± 0.25	4.03 ± 0.37	0.045	C > A
	Environmental	3.15 ± 0.21	4.58 ± 2.02	7.20 ± 0.78	8.45 ± 0.64	0.009*	D > A, D > B
European rabbit	Physical	1.10 ± 0.14	1.73 ± 0.46	1.22 ± 0.39	2.48 ± 0.64	0.021*	D > C
	Psychological	3.00 ± 0.42	2.98 ± 0.59	3.45 ± 0.45	3.70 ± 0.40	0.128	A = B = C = D
	Procedure	3.60 ± 0.00	3.63 ± 0.37	3.88 ± 0.34	3.03 ± 0.60	0.140	A = B = C = D
	Environmental	5.00 ± 0.99	5.00 ± 0.85	6.78 ± 0.75	8.07 ± 0.55	0.007*	D > B

Three inspectors evaluated scores for 11 animal species from 16 zoos and calculated mean scores. There were three species that showed significant differences according to zoo grade: black-tailed prairie dogs, Meerkats, and European Rabbit (*p* < 0.05). The zoo levels were defined as (A) (AZA-accredited zoos), (B) (KAZA-certified zoos), (C) (large petting zoos, >50 species, >1,000 individuals, >3,000 m^2^), or (D) (small petting zoos). The zoo levels were defined as (A) (AZA-accredited zoos), (B) (KAZA-certified zoos), (C) (large petting zoos, >50 species, >1,000 individuals, >3,000 m2), or (D) (small petting zoos). These results highlight the disparities in welfare conditions across different zoo grades, suggesting that lower-grade zoos may have poorer welfare standards for these species. * indicates a statistically significant difference.

The Kruskal-Wallis test and Dunn’s post-hoc test were also used to analyze the differences in environmental variable scores among the groups. The analysis of environmental variables revealed statistically significant differences in illuminance, temperature, and humidity across Groups A-D (Table 3). Specifically, post-hoc analyses indicated that: Illuminance in Group B was significantly higher than in Groups A, C, and D (*p* = 0.001). Temperature in Group B was significantly higher than in Groups C and D (*p* = 0.001). Humidity in Groups A and C was significantly higher than in Groups B and D (*p* = 0.001) (see Table 4).

**Table 4:** The environmental parameters differences among groups A to D.

Items	A	B	C	D	*p*	Post hoc
Sound	56.01 ± 5.84	59.81 ± 12.44	59.10 ± 9.40	58.21 ± 7.30	0.669	-
Illuminance	2331.18 ± 2495.62	17608.49 ± 41199.67	1850.05 ± 3333.37	3781.63 ± 4894.53	0.001	B > A = C=D
Temperature	26.65 ± 1.67	27.79 ± 5.09	25.22 ± 1.26	25.27 ± 2.07	0.001	B > C=D
Humidity	72.82 ± 3.37	73.14 ± 46.55	73.97 ± 7.65	66.14 ± 10.02	0.001	-
Odor	46.29 ± 176.93	83.79 ± 193.14	104.65 ± 233.56	43.91 ± 181.13	0.081	-

This table shows the differences in environmental parameters (sound, illuminance, temperature, relative humidity, and odor) among zoo grades (A to D). Three inspectors evaluated scores for 11 animal species from 16 zoos and calculated mean scores. The zoo levels were defined as (A) (AZA-accredited zoos), (B) (KAZA-certified zoos), (C) (large petting zoos, >50 species, >1,000 individuals, >3,000 m^2^), or (D) (small petting zoos). The zoo levels were defined as (A) (AZA-accredited zoos), (B) (KAZA-certified zoos), (C) (large petting zoos, >50 species, >1,000 individuals, >3,000 m^2^), or (D) (small petting zoos). This data suggests that environmental conditions vary significantly between zoo grades and that these variations could impact animal welfare differently. * indicates a statistically significant difference.

Table 5 presents the correlations between environmental variables (e.g., sound, illuminance, temperature, humidity, and odor) and M-AWAG factors (e.g., general condition, clinical assessment, feces, activity, food and drink intake, and training). Several statistically significant but weak associations were identified. Food and drink intake was positively associated with sound (r = 0.28, *p* < 0.001) and negatively associated with illuminance (r = −0.24, *p* = 0.007) and relative humidity (r = −0.17, *p* = 0.046). Activity was also negatively associated with relative humidity (r = −0.18, *p* = 0.042).

**Table 5:** The correlation coefficients between environmental parameters and AWAG factors.

Environmental parameters	General condition	Clinical assessment	Feces	Activity	Food & drinks	Training
	r(*p*)	r(*p*)	r(*p*)	r(*p*)	r(*p*)	r(*p*)
Sound	0.10 (0.245)	0.11 (0.215)	0.08 (0.342)	0.09 (0.286)	0.28^*^ (<0.001)	−0.06 (0.473)
Illuminance	0.15 (0.094)	0.13 (0.135)	0.09 (0.348)	−0.05 (0.568)	−0.24^*^ (0.007)	0.07 (0.463)
Temperature	0.06 (0.509)	0.04 (0.625)	0.01 (0.895)	−0.09 (0.290)	−0.16 (0.059)	0.01 (0.950)
humidity	−0.15 (0.076)	−0.15 (0.076)	0.08 (0.338)	−0.18^*^ (0.042)	−0.17^*^ (0.046)	−0.07 (0.442)
Odor	−0.02 (0.796)	−0.02 (0.796)	−0.11 (0.237)	0.14 (0.136)	−0.10 (0.285)	−0.01 (0.574)

This table presents the correlation coefficients between environmental parameters (sound, illuminance, temperature, relative humidity, odor) and AWAG factors (general condition, clinical assessment, feces, activity, food and drinks, training). A significant positive correlation was found between sound and food/drinks (r = 0.28, *p* < 0.001). Illuminance had a significant negative correlation with food/drinks (r = −0.24, *p* = 0.007). Relative humidity showed significant negative correlations with activity (r = −0.18, *p* = 0.042) and food/drinks (r = −0.17, *p* = 0.046). These findings suggest that specific environmental factors, such as sound and humidity, may significantly influence animal activity levels and feeding behavior, highlighting the importance of optimizing environmental conditions to improve animal welfare. * indicates a statistically significant difference.

These findings suggest that some environmental conditions may be related to feeding behavior and activity patterns; however, because the present study was observational in design and the correlation coefficients were weak, these results should be interpreted cautiously as associations rather than causal effects. Temperature and odor were not significantly associated with the M-AWAG factors within the measured ranges.

In summary, the study found that welfare scores were generally poorer in lower-rated zoos (Group D) compared to higher-rated ones (Groups A and B), particularly for species such as black-tailed prairie dogs, Meerkats, and European rabbits. Environmental factors such as illuminance, temperature, and humidity significantly influenced animal welfare, highlighting the importance of optimal environmental management in maintaining high welfare standards in zoos.

## 4 Discussion

### 4.1 Assessing animal welfare in farms and zoos: a comparative overview

Research on farm animal welfare has been extensive and systematic, aiming to enhance animal well-being while simultaneously increasing economic benefits (22, 23, 25). Scholars have focused on various environmental factors to maximize animal health and productivity, thereby contributing to optimal rearing environments that improve both welfare and efficiency (14, 51, 52). Notably, the Welfare Quality^®^ (WQ) protocol is a widely used method for assessing farm animal welfare, encompassing both physical and micro-environmental elements (15, 53, 54). Physical environmental factors include flooring, bedding, outdoor exposure areas, and the size and structure of rearing spaces, while micro-environmental factors encompass temperature, humidity, illuminance, and airborne bacterial concentrations (22, 55, 56). Comprehensive environmental assessments enable precise measurement of factors directly affecting animal health and welfare, facilitating efforts to create ideal rearing conditions (34, 53). Furthermore, the WQ protocol integrates animal-based indicators—such as physical condition, behavior, and psychological state—with resource-based indicators like the quality and quantity of feed and water and the adequacy of rearing facilities (26, 53). This holistic approach ensures that both external environmental conditions and internal animal responses are considered, providing a thorough assessment of welfare (26, 33, 34). Precision instruments such as thermometers, hygrometers, and light meters are employed to evaluate micro-environmental factors, offering objective and quantitative assessments of how these conditions influence productivity, growth rates, reproductive success, and disease prevalence (22, 55, 57). Additionally, welfare assessments for farm animals are typically conducted at the group level, assuming uniform environmental conditions within groups, which allows for efficient evaluation and management in large-scale operations (53, 58).

In contrast, animal welfare research in zoos employs distinct assessment approaches due to the diversity of species and their individual needs (26, 33, 34). Zoos house a wide range of species, each with unique ecological and behavioral requirements, necessitating individualized welfare assessments that account for their specific physical and psychological needs (6, 26, 32). According to the AZA^9^, there are at least 10,000 species registered in zoos globally, and the assessments specifically target these animal species (59, 60). A primary tool utilized in zoos is the AWAG (7, 31, 32). AWAG evaluates animals based on parameters including physical condition, behavior, psychological state, environmental factors, and procedural elements (32). By employing a quantified scoring system, AWAG systematically analyzes the welfare levels of individual animals, enabling tailored management practices (7, 26, 32). Within the AWAG framework, environmental assessments involve detailed analyses of enclosure size and structure, facility design, safety, cleanliness, temperature, humidity, illuminance, sound levels, and the provision and effectiveness of environmental enrichment (32). These assessments provide comprehensive insights into how living conditions impact animal welfare. However, AWAG and other existing assessment tools exhibit significant limitations in exclusively evaluating environmental factors. While AWAG integrates environmental measures as part of a broader welfare assessment, there is a lack of specialized tools dedicated solely to environmental assessments within zoos (26, 31, 35). This gap implies that critical environmental parameters may not be thoroughly evaluated, potentially overlooking subtle yet impactful factors affecting welfare (5, 14, 55). For instance, precise measurements of air quality, continuous monitoring of temperature and humidity fluctuations, and detailed assessments of sound pollution often require advanced technologies and specialized expertise not fully addressed by existing frameworks (8, 14, 55).

Moreover, many zoo assessment tools rely heavily on subjective judgments by keepers and experts, which can introduce biases and inconsistencies (34, 35). The Animal Welfare Risk Assessment Process (AWRAP), for example, employs keeper-based ratings that may vary significantly depending on individual perceptions and experiences (26, 61). Similarly, the Zoological Information Management System (ZIMS), while facilitating global information sharing and data storage, faces challenges in consistent implementation across venues with limited resources and in non-English-speaking regions, complicating standardized environmental assessments (1, 26). The Welfare Discussion Tool (WDT) and WelfareTrak further highlight challenges in environmental evaluations. While WDT ensures inter-rater reliability and promotes management changes through regular discussions, it remains time and resource-intensive, particularly for facilities with constrained budgets (26, 27, 33). WelfareTrak’s quantitative scoring system, although effective in tracking changes over time, often lacks comprehensive integration with animal-based measures, leading to potential gaps in environmental evaluations (26). These deficiencies underscore the need to develop standardized environmental assessment indicators and methodologies tailored specifically for zoos (3, 6, 26).

### 4.2 Legal reforms and the implementation of the inspector system in South Korea

Internationally, inspector systems have been actively implemented to enhance animal welfare in zoos (38, 47). For example, the United Kingdom’s Zoo Licensing Act 1981 (62) mandates that all zoos obtain licenses from local authorities and undergo regular inspections by appointed inspectors (47). These inspectors assess animal welfare standards, conservation efforts, and educational activities to ensure high levels of animal care. Similarly, the European Union’s Directive 1999/22/EC (63) requires member states to fulfill conservation and animal welfare requirements through the licensing and regular inspection of zoos. In the United States, the Animal and Plant Health Inspection Service (APHIS) under the United States Department of Agriculture (USDA) conducts unannounced inspections of zoos (64), focusing on facilities, veterinary care, and animal handling practices in accordance with the Animal Welfare Act (65). These international examples demonstrate the critical role that standardized inspections and regulatory oversight play in maintaining and improving zoo animal welfare. These foreign inspector systems have significantly influenced South Korea’s zoo management policies. Specifically, the UK’s zoo licensing and inspection system, along with the EU’s directives, have been referenced in the enactment of South Korea’s Zoo and Aquarium Management Act, directly impacting the establishment of animal welfare standards and the introduction of an inspector system (31, 37, 38). Consequently, South Korea has strengthened its regulations by incorporating these international examples of animal welfare improvement (31).

The amended Zoo and Aquarium Management Act, which entered into force on October 1, 2025 (36), provides an essential legal framework to structure the operation of zoos and aquariums and to enhance animal welfare. This law transitions from a registration system to a licensing system, thereby reinforcing the establishment and operation standards of zoos and aquariums. Furthermore, by introducing an inspector system that conducts regular evaluations and unannounced inspections, it establishes a structure for continuous monitoring and improvement of animal welfare. The introduction of the inspector system is expected to significantly contribute to improving animal welfare standards (38, 47). According to the guidelines (66), inspectors—qualified professionals defined by presidential decree—are tasked with regularly inspecting zoo facilities, living environments, and the welfare status of animals. Recognizing that securing the expertise of inspectors is crucial, the regulations require them to have at least 5–10 years of relevant experience in fields such as veterinary medicine, animal management, or zoo operation. This requirement ensures that inspectors can thoroughly and accurately evaluate zoo facilities and animal welfare conditions. Such professionalism enables reliable assessments that contribute to enhancing animal welfare and is essential for the effective implementation of the inspector system. In addition to their expertise, inspectors are mandated to use standardized forms such as on-site inspection sheets, recording “Yes/No” checklists and the grounds for their judgments. This standardization helps maintain consistency in inspection results and minimizes subjective bias, thereby increasing transparency and objectivity in the inspection process. Systematic documentation ensures the clarity and transparency of each inspection, playing a vital role in enhancing public trust.

To further maintain the objectivity and reliability of the inspector system, measures to prevent conflicts of interest are included. Specifically, if an inspector or their family has an interest in the zoo being inspected, the inspector must be excluded from the evaluation. This policy ensures fairness in the inspection process and maintains public trust, thus enhancing the credibility of the inspector system. Additionally, inspectors are required to continuously monitor the zoo’s improvement status after evaluations and to conduct additional inspections if necessary, ensuring that animal welfare levels are consistently maintained and improved. Apart from these regulatory measures, voluntary participation by zoos and raising public awareness are also important aspects of improving animal welfare. The guidelines recommend acknowledging and encouraging zoos that practice excellent animal welfare standards, thereby promoting voluntary participation and motivating other zoos to elevate their welfare levels. Such recognition systems can contribute to enhancing overall societal animal welfare by increasing public awareness and encouraging visitors to choose zoos based on animal welfare standards. Furthermore, the clear assignment of responsibility and strengthened authority are among the key advantages of the guidelines. Inspectors are granted the authority to issue correction orders or recommend improvements upon discovering legal violations or factors detrimental to animal welfare. If necessary, they can also recommend administrative actions such as suspension of operations or license revocation, thereby exerting a practical effect in improving and maintaining animal welfare. In addition, continuous education and training are vital for maintaining and enhancing the professionalism of inspectors. The guidelines emphasize that inspectors should receive ongoing education and training to acquire the latest animal welfare guidelines and animal management techniques, aligning with evolving international animal welfare standards. This commitment to professional development allows inspectors to provide better evaluations and results, thereby contributing to the continuous advancement of animal welfare levels.

### 4.3 Integrating M-AWAG and environmental metrics: a dual-assessment model for Korean zoos

In this study, we employed a Modified Animal Welfare Assessment Grid (M-AWAG) to quantitatively evaluate the level of animal welfare in Korean zoos (21, 32). The M-AWAG served as a crucial assessment tool that numerically represents the condition of animals by comprehensively considering their health, behavior, and environment (7, 35) (See the modified AWAG in Supplementary Tables 1–4). Although this evaluation method is time-consuming, it is essential to conduct assessments based not only on environmental factors (e.g., temperature, humidity, sound) but also on the animals’ own conditions (e.g., behavior, health status) (31, 35). However, there are insufficient resources and budget to apply a comprehensive animal-based evaluation to all species in all zoos across Korea (35, 40). To address these limitations, this study conducted additional environmental assessments alongside the M-AWAG. While the M-AWAG itself includes elements of environmental evaluation, supplementary assessments were carried out to perform more detailed and objective measurements of external factors such as temperature, humidity, and sound levels. This dual approach enabled thorough welfare evaluations even in situations where extensive animal-based assessments across all species were not feasible due to resource constraints.

This dual approach allowed for the efficient utilization of resources and budget in several ways. First, by using the basic evaluation tools of the M-AWAG to rapidly assess the overall welfare status of animals, we reduced the time required for the entire assessment (31, 35). The additional environmental evaluations allowed us to focus on the most critical external factors, effectively allocating limited resources and budget to key elements (21, 55). This prevented the wastage of resources on unnecessary assessment items and enabled concentration on factors that have the most significant impact on animal welfare. Furthermore, while the M-AWAG focuses on the animals’ behavior and health status, the additional environmental evaluations provided detailed measurements of physical environmental conditions, allowing the two assessment methods to function complementarily (22, 31). For instance, after assessing the animals’ welfare status using the M-AWAG, we could specifically identify the causes by measuring temperature and humidity. This complementary data collection avoided redundant assessments and contributed to a more comprehensive and accurate understanding of the welfare status. In addition, the supplementary environmental evaluations collected data using standardized measurement tools and procedures, thereby enhancing the consistency and reliability of the assessments (15, 53, 54). This approach enabled evaluations to be conducted using the same criteria across different species and facilities, minimizing variability that could occur during the assessment process (32, 35). The use of standardized tools reduced errors due to differences in evaluator proficiency and streamlined data collection and analysis processes, saving time and costs (7, 26, 34). Moreover, by prioritizing the most influential environmental factors and limiting the scope of assessments—rather than conducting comprehensive evaluations for all species and facilities—we could use resources and budget more efficiently. This strategy allowed us to manage important welfare indicators without neglecting them, even within limited resources, and increased the focus of the assessments to derive more effective welfare improvement measures (26, 35).

Additional advantages gained by combining the M-AWAG with goal-oriented environmental evaluations include improvements in objectivity and precision. While the M-AWAG integrates both animal-based and resource-based indicators, environmental evaluations offer more detailed and objective measurements of specific external conditions, reducing reliance on subjective judgments inherent in M-AWAG assessments and enhancing the overall objectivity and precision of the evaluations (7, 31, 35). This increases the reliability of the assessment results and enables a more accurate understanding of the welfare status (34, 55). Lastly, independent environmental evaluations helped mitigate potential biases and inconsistencies that could arise from the subjective judgments inherent in M-AWAG assessments, playing a crucial role in maintaining the fairness and objectivity of the evaluations (6, 21, 26). This study thus demonstrates that this dual approach not only enables efficient use of resources and budget but also contributes to enhancing the accuracy and reliability of animal welfare assessments. It provides a practical methodology for effective animal welfare management and improvement even within limited resources and will serve as important foundational data for the future development of zoo policies in South Korea.

### 4.4 Interpretation of results: evaluating welfare outcomes

A total of 16 zoos were evaluated to determine the welfare status of their animals. The primary objective was to analyze differences in animal welfare across different zoo groups and to elucidate the impact of zoo types and operational practices on animal welfare.

#### 4.4.1 Statistical differences in M-AWAG scores by species

To effectively analyze the welfare status of the animals, the 16 South Korean zoos were categorized into four groups (A, B, C, D) based on their accreditation status, the number and species of animals they housed, and their overall area (Table 1). Group A comprised large zoos accredited by the AZA, Group B included large zoos accredited by the KAZA, Group C consisted of large uncertified zoos (housing over 50 species and more than 1,000 animals), and Group D included small uncertified zoos (housing fewer than 50 species and fewer than 1,000 animals). Welfare assessments were conducted for 11 species that were housed in most zoos across each group. Utilizing the Kruskal-Wallis test and Dunn’s post-hoc test, the results indicated that the welfare levels of black-tailed prairie dogs (*Cynomys ludovicianus*), meerkats (*Suricata suricatta*), and European rabbits (*Oryctolagus cuniculus*) housed in Group D zoos were generally lower compared to those in Group A or B zoos (Table 3; Supplementary Table 5). Furthermore, when comparing the average M-AWAG scores across four categories for the 11 species, it was found that environmental scores, psychological scores, and procedural scores were higher than physical scores, displaying a consistent pattern across all species (e case of black-tailed prairie dogs), Group D zoos recorded higher scores in the psychological and environmental categories compared to Group A or B zoos (*p* < 0.05) (Table 3; Supplementary Table 5). The elevated scores in these categories indicate poorer welfare conditions. The rise in popularity of exotic wildlife experiences in Korea has led to a rapid increase in small indoor zoos classified under Group D. During this expansion, indiscriminate interactive activities and substandard housing conditions have been identified as key factors undermining animal welfare (31, 35, 40). In Group D facilities, black-tailed prairie dogs are often kept in small enclosures with cement floors surrounded by glass, housing only one or two individuals for interaction purposes. The black-tailed prairie dog is diurnal, highly active throughout the year, and maintains complex social relationships (67). In the wild, they exhibit natural behaviors such as foraging from sunrise to sunset, digging burrows, and living in colonies (68). However, in these facilities, the ability of black-tailed prairie dogs to express their instinctive behaviors is severely restricted, leading to increased stress and the emergence of abnormal behaviors, such as repetitive pacing and excessive digging within confined spaces.

For meerkats, Group D zoos scored higher in the physical category compared to Group A or B zoos (*p* < 0.05) (Table 3; Supplementary Table 5). Most Group D zoos offer visitor participation activities for meerkats, which, despite being carnivorous, receive vegetables or excessive amounts of food, resulting in obesity and nutritional imbalances. In contrast, Group A zoos enhance meerkat welfare by providing various behavioral enrichment items such as insects, food puzzles, and numerous toys. These enrichment activities are crucial for meerkats, a species known for their high social and cognitive needs (69). However, in Group D zoos, meerkats are often housed in cement-floored enclosures surrounded by glass walls. Newly hired managers in these facilities frequently fail to recognize the necessity for environmental improvements, leading to inadequate enrichment and suboptimal living conditions. Consequently, the welfare scores for meerkats declined, and severe abnormal behaviors, such as excessive grooming and stereotypic movements, emerged. This decline highlights the sensitivity of meerkats to their living environments and underscores the importance of providing appropriate physical and psychological stimuli to meet their complex behavioral needs. Additionally, Group D zoos recorded significantly higher scores in the environmental category compared to Group A or B zoos (*p* < 0.05), and higher scores in the psychological category compared to Group A zoos (*p* < 0.05). Since the early 2000s, the concept of behavioral enrichment has been introduced in Korean zoos, but most zoos outside of large institutions (Group A or B) have failed to provide sufficient behavioral enrichment for their animals. The lack of adequate enrichment in smaller, uncertified zoos directly impacts the psychological well-being of meerkats, a species that thrives in stimulating and socially interactive environments (5, 29, 69). In the procedural category, Group C zoos scored higher than Group A zoos (*p* < 0.05), and visitor scores also varied across zoos in the procedural domain. Visitor numbers affect the welfare of sensitive species such as birds and mammals, including meerkats. Increased visitor numbers lead to higher sound levels, which can influence the behavior and physiological states of mammals (1, 2, 4). Although Group C or D zoos may have fewer daily visitors compared to Group A or B zoos, the closer proximity between visitors and animals can have a more substantial impact on welfare (12, 69). For meerkats, frequent exposure to high sound levels can cause stress, disrupt social interactions, and reduce overall well-being (11, 12, 69).

Regarding European rabbits, Group D zoos scored higher in the physical category compared to Group A or B zoos (*p* < 0.05) (Table 3; Supplementary Table 5). Similar to other animals, European rabbits in Group D zoos were frequently used in interactive activities, resulting in a high incidence of obesity and nutritional imbalances. Prior to the enactment of relevant laws, there were no guidelines for feeding animals, allowing visitors to feed them freely throughout the day (35, 40). This lack of regulation led to excessive and unbalanced diets, contributing to the physical health decline of the European rabbits. In the environmental category, Group D zoos also scored higher than Group B zoos (*p* < 0.05). Indoor enclosures in Group D zoos lacked adequate soil for digging and housed European rabbits in groups of one or two, which is inconsistent with their natural social structures. European rabbits are social animals that thrive in larger groups and require environments that allow for digging and burrowing (2, 35). The restrictive housing methods employed in Group D zoos limit these natural behaviors, leading to increased stress and the development of abnormal behaviors such as overgrooming and reduced social interactions (1, 4, 5). The inadequate provision of space and environmental enrichment in Group D zoos not only impairs the European rabbits’ ability to perform natural behaviors but also adversely affects their physical and psychological health (5, 29). The high levels of obesity observed in these European rabbits are indicative of poor dietary management and insufficient opportunities for physical activity, further compromising their overall welfare. These findings highlight the necessity for improved housing conditions and regulated feeding practices in smaller, uncertified zoos to ensure that European rabbits can maintain their natural behaviors and achieve optimal health and well-being.

#### 4.4.2 The correlation between environmental parameters and M-AWAG factors

The sensory environment of an animal plays a crucial role in its overall well-being. In this study, we investigated the correlations between environmental parameters and animal welfare indicators as measured by the M-AWAG factors. First, Higher sound levels in zoo enclosures were found to correlate with higher scores in the ‘Food & Drink Intake’ parameter, indicating that increased sound was associated with reduced food and water intake (Table 5). This finding aligns with previous research demonstrating that anthropogenic sound can elicit stress responses in animals. For instance, David et al. observed that captive anteaters near construction sites experienced weight loss due to heightened activity and increased sound levels (11). They emphasized that continuous sound assessment is essential in animal care operations, as sound significantly affects animal well-being. Mitigating the impact of sound can be achieved by reducing sound sources or using barriers to dampen sound transmission. Similarly, Richard et al. assessed the responses of elephants, giraffes, emus, and crocodiles to construction sound during a major building project at a New Zealand urban zoo (70). Their results showed that giraffes, elephants, and emus exhibited behaviors indicative of stress or agitation, such as heightened vigilance and increased locomotion. These animals also preferred quieter areas of their enclosures during sound exposure, indicating the adverse effects of sound on animal welfare.

Beyond construction-related sound, zoo animals are routinely exposed to large numbers of visitors, with the associated sound levels identified as significant contributors to overall sound within these facilities (12). Paul et al. found that higher visitor numbers and louder sound led to increased vigilant and vocal behaviors in birds housed in both urban and rural zoos (13). Factors such as husbandry activities, visitor presence, and the proximity of surrounding species all influence animal welfare. Therefore, an animal’s behavioral response to changing acoustic environments can provide valuable insights into how its welfare is affected (13). In domestic petting zoos, multiple species are often housed together in confined spaces, making them susceptible to stress from sound generated by other animals or loud music. Animals housed in glass enclosures may be relatively protected from artificial sound, whereas those in non-glass enclosures may experience significant stress due to ambient sound. Currently, there are no established guidelines for acceptable sound levels for animals in zoos and aquariums, both domestically and internationally (11, 12). Establishing such guidelines would be instrumental in protecting animals from sound-induced stress.

Temperature and humidity are well-known to significantly impact the productivity and health of farm animals, leading to extensive research in agricultural settings (22, 51, 57). However, few studies have examined the impact of these microclimate factors on animal welfare in zoos (8–10). Microclimate is defined as the totality of physical (temperature, humidity, airflow), chemical (toxic gases) and biological (bacteria, viruses, fungi) factors that, by their continuous or simultaneous action, have a decisive influence on animal health and production (55). Zoo microenvironments vary according to geographical climate and are significantly influenced by the physiological characteristics of the housed animals (8, 19). In our study, while temperature did not show significant correlations with M-AWAG factors within the measured ranges, it remains a critical factor influencing thermal comfort and overall health. Proper temperature management is essential, although in this study, temperature may have been adequately controlled in the zoos assessed, or its impact on the specific welfare indicators measured may be less direct. Relative humidity, on the other hand, showed significant correlations with welfare indicators (6, 7, 26). Higher humidity levels were negatively correlated with both ‘Food & Drink Intake’ (r = −0.17, *p* = 0.046) and ‘Activity’ levels (r = −0.18, *p* = 0.042), implying that excessive humidity may create discomfort or lethargy, reducing animals’ inclination to engage in normal feeding and activity patterns. The variations in humidity among zoo groups may be due to differences in enclosure design or maintenance practices affecting moisture levels.

Research on the effects of illuminance levels on zoo animal welfare is limited, with most studies focusing on zoo architecture and enclosure design (5, 29, 55). In our study, significant differences in illuminance were observed among different zoo groups. Group B had significantly higher illuminance levels compared to other groups, possibly due to adherence to specific lighting standards or facility designs that maximize natural light exposure. Higher illuminance levels were associated with lower scores on the ‘Food & Drink Intake’ parameter (r = −0.24, *p* = 0.007), suggesting that adequate lighting promotes better feeding behavior by improving visibility and simulating natural conditions. Exhibits with outdoor enclosures or access to direct sunlight had higher illuminance levels, which may enhance animals’ circadian rhythms and overall well-being. Conversely, interactive indoor zoos tended to have lower illuminance due to interior design factors. Permanently housing animals indoors with insufficient lighting can be detrimental to their health and welfare, underscoring the need for illuminance regulations in the licensing of indoor zoos.

The significant correlations between environmental variables and welfare indicators emphasize the necessity of meticulous environmental management in zoos—including controlling sound levels, providing adequate lighting, and maintaining optimal humidity. These results underscore the importance of individualized welfare assessments and species-specific environmental management practices. The significant differences in environmental conditions among zoo groups highlight the potential benefits of accreditation and adherence to standardized welfare protocols. Accredited zoos may have more stringent guidelines and resources to optimize environmental conditions, thereby enhancing animal welfare. In conclusion, implementing standardized welfare assessments and adopting best practices in environmental management can significantly improve the well-being of captive animals, contributing to the sustainable development of the zoo industry and aligning facilities with international animal welfare standards.

These group-level differences should be interpreted cautiously, as zoo classification in this study reflected multiple contextual factors in the Korean setting, including accreditation status, operational type, and size, rather than a single isolated variable.

### 4.5 Policies and management strategies for improving zoo animal welfare

The low welfare levels and inadequate environmental management observed in Group D zoos in this study highlight a significant potential for improvement through the implementation of new legislation and the introduction of an inspector system. Inspectors can enhance animal welfare by evaluating and recommending necessary improvements for managing environmental variables such as temperature, humidity, sound, and lighting (47). The emphasis on microenvironmental management and the provision of housing tailored to the specific characteristics of each species, as identified in this study, can be incorporated into legally mandated facility standards and upgrades (8, 22, 55). By utilizing their professional expertise, inspectors can scientifically assess environmental factors affecting animal welfare and propose concrete corrective measures (35, 47).

#### 4.5.1 Based on the findings of this study, we propose several policy and management strategies to improve zoo animal welfare

First, it is essential to regularly monitor and appropriately adjust microenvironmental factors such as sound, lighting, temperature, and humidity to reduce stress and discomfort in animals. Implementing measures like installing soundproofing facilities, ensuring access to natural lighting, and maintaining appropriate temperature and humidity levels can significantly enhance animal welfare (2, 5, 29).

Second, providing housing environments that meet the ecological and behavioral needs of each species is crucial. For instance, black-tailed prairie dogs and meerkats require environments that support digging and social living, necessitating sufficient space and appropriate facilities. Interactive activities with visitors should be carefully managed to prevent negative impacts on animal welfare and should be conducted under the supervision of trained caretakers. Specifically, feeding by visitors should be restricted to prevent nutritional imbalances.

Third, it is important to station veterinarians and animal behaviorists with specialized knowledge of animal welfare in zoos and to enhance awareness among caretakers and managers through regular training programs. Utilizing standardized assessment tools like the M-AWAG for regular welfare evaluations and monitoring can help identify areas needing improvement and facilitate the implementation of appropriate measures. Strengthening legal standards for zoo animal welfare and establishing systems to monitor and enforce compliance can further enhance overall welfare levels. Additionally, promoting research and data sharing is vital for advancing zoo animal welfare studies. Accumulating scientific evidence for welfare improvements by sharing domestic and international case studies and data can inform best practices.

Finally, providing alternative solutions for animals affected by the closure of wild animal cafes and indoor zoos is essential. This could include establishing sanctuaries or rehabilitation centers where animals can receive proper care and, when possible, be reintroduced to their natural habitats. Offering support and guidance to business owners for transitioning to more welfare-friendly models can also mitigate negative impacts.

Implementing these comprehensive policies and management strategies can enhance the quality of life for zoo animals and provide visitors with more meaningful and educational experiences. This approach aligns zoos with international animal welfare standards and contributes to the sustainable development of the Korean zoo industry.

### 4.6 Limitations of the study

This study evaluated the welfare status of zoo animals in South Korea by utilizing the Modified Animal Welfare Assessment Grid (M-AWAG) alongside microenvironmental assessments. Despite its contributions, several limitations must be acknowledged.

Firsts, the study was conducted over a short period from July to August 2020, which does not account for seasonal variations or long-term changes in welfare levels. Additionally, microenvironmental variables were measured at specific points in time, potentially overlooking fluctuations related to different times of day or seasons. For instance, temperature, humidity, and sound levels can vary significantly depending on the time of day or season. Given that animal welfare can be influenced by seasonal and long-term environmental changes, long-term monitoring and seasonal assessments are necessary to obtain a more comprehensive understanding of welfare status (8, 35, 61).

Second, this research was conducted before the implementation of the inspector system, thereby not reflecting the effects of recent legal amendments or institutional changes currently in place. As a result, the findings may differ from the current welfare conditions of zoos. Future studies should incorporate the latest legal standards and institutional changes to provide an updated evaluation of animal welfare.

Third, the methodology of integrating M-AWAG with microenvironmental assessments was novel in the South Korean context, necessitating further validation of this combined assessment approach. Additional studies are required to confirm the validity and reliability of this integrated method. Specifically, evaluating its applicability across a broader range of species and varying environmental conditions will help refine the assessment tools, enabling more precise and accurate welfare evaluations.

Fourth, while the M-AWAG includes behavioral indicators to assess psychological welfare, it does not incorporate animal-based physiological measurements such as hormonal levels, which can provide objective data on the animals’ stress and overall health. Psychological welfare was evaluated through observed behaviors rather than precise quantitative metrics, potentially limiting the depth and objectivity of these assessments. The absence of physiological data means that internal states of the animals, which are not always externally observable, may not be fully captured (6, 7, 26). Future research should integrate physiological indicators, such as cortisol levels, to complement behavioral assessments and offer a more comprehensive evaluation of animal welfare.

To address these limitations, ongoing and long-term research is essential. Enhancing and standardizing assessment tools, expanding the range of zoos and species included in studies, implementing continuous environmental monitoring, incorporating animal-based physiological measurements, and reflecting the latest laws and institutional changes will enable a more accurate and comprehensive evaluation and improvement of animal welfare levels.

## Data Availability

The original contributions presented in the study are included in the article/Supplementary material, further inquiries can be directed to the corresponding author.
