# Correction: A study of animal welfare in 11 species across 16 Zoos using M-AWAG and its correlations with environmental variables

**DOI:** 10.3389/fvets.2026.1975237

**Published:** 2026-09-25

**Authors:** Hyejin Kang

**Affiliations:** Department of Horse/Companion and Wild Animal Science, Kyungpook National University, Daegu, Republic of Korea

**Keywords:** animal welfare assessment, animal welfare assessment grid, microenvironment, South Korea, zoo animal welfare, zoo animals, zoo welfare inspection

Author Seung Aee Ma was erroneously included as an author in the published article. The correct author list reads:

Hyejin Kang^*^

Department of Horse/Companion and Wild Animal Science, Kyungpook National University, Daegu, Republic of Korea

Author Seung Aee Ma was erroneously assigned as corresponding author.

The correct corresponding author is Hyejin Kang.

Hyejin Kang, animalwelfare@snu.ac.kr

The original version of this article has been updated.

