# Supplementary material for: A study of animal welfare in 11 species across 16 Zoos using M-AWAG and its correlations with environmental variables
Source: Front Vet Sci. 2026 May 19;13:1814857. doi: 10.3389/fvets.2026.1814857 (PMC13225989; doi:10.3389/fvets.2026.1814857)
Supplement: Supplementary file 1 [file Table_1.DOCX]

**Supplementary Table 1. Modified Animal Welfare Assessment Grid: Welfare Scoring for Physical Sections**

| General condition | | Clinical assessment | | Faeces | | Activity | | Food and drinks | |
| --- | --- | --- | --- | --- | --- | --- | --- | --- | --- |
| Weight, condition, coat/feather condition | Score | Injury, alopecia, vomiting and other clinical symptoms | Score | Faecal consistency | Score | Activity level, mobility | Score | Not eating/drinking or reported hungry/thirsty | Score |
| Weight within normal range and good condition and coat/feather condition. | 1 | Clinically healthy; no injuries | 1 | formed | 1 | Normal | 1 | Normal | 1 |
| Weight outside normal range by <10%. And/or a minor reduction in coat/feather condition | 3 | Mild transient subclinical/clinical  Symptoms, minor injury | 3 | Soft | 3 | Increased activity or slight reduction in activity | 3 | Food/water intake reduced,  or reported hungry for two  to three days | 3 |
| Weight outside normal range by<20%. r a substantial  reduction in coat/feather  condition. | 5 | Mild-to-moderate clinical disease | 5 | Loose | 5 | Sizeable increase or  decrease in activity that  shows some recovery | 5 | Food/water intake reduced for 1~2 days or reported hungry for 2~6 days | 5 |
| Weight outside normal range by  <30% | 7 | Moderate clinical disease or injury | 7 | Loose | 7 | Sizeable increase or  decrease in activit | 7 | Food/water intake reduced  or reported hungry for more  than 7days | 7 |
| Weight outside normal range by  >30% | 9 | Severe disease with clinical symptoms  or severe injury | 9 | Very loose | 9 | Minimal movement, Lethargy or signs  of hyperactivity | 9 | Animal reported very thirsty  or dehydrated for 1~2 day  or anorexic for 1~2 day | 9 |
| Weight outside normal range by  >30% and over/under condition  for >21 days. | 10 | Severe disease with clinical signs and/  or severe injury, and no chance of full or  moderate recovery | 10 | Soft to very loose  with the presence  of blood and/or  mucus. | 10 | Complete lethargy | 10 | Animal reported very thirsty  or dehydrated for three days  or anorexic for three days | 10 |

**Supplementary Table 2. . Modified Animal Welfare Assessment Grid: Welfare Scoring for Psychological Sections**

| Abnormal behaviours | | Response to catching event | | Social status | | Enrichment | | Aversion to ‘normal’ events | | Training | |
| --- | --- | --- | --- | --- | --- | --- | --- | --- | --- | --- | --- |
| Automutilation, regurgitation, stereotypies | S* | Trained/ habituated, catching method | S | Hierarchy upset/  dispute, aggression/  bullying | S | Includes food-based enrichment | S | Trained/ habituated, emotional expression | S | Expression, participation | S |
| None | 1 | Trained/habituated animal, no  stress or fear | 1 | None | 1 | Multiple types/items of enrichment provided, all used well | 1 | Trained/  habituated  animal, none  noted | 1 | Animal participated well  with the session | 1 |
| Low~medium frequency, minimal  time spend, no damage done | 3 | Trained/habituated animal, some stress or fear noted; caught without intervention;  possible multiple attempts | 3 | Some present in the  group, short duration, | 3 | Multiple enrichment provided, some  used well | 3 | Trained/  habituated  animal, some  stress or fear  noted | 3 | Some stress or fear noted or no  training took place | 3 |
| Medium~high frequency, some  time spend, no damage done | 5 | Non-habituated animal, stress or fear noted; caught by crush  or box | 5 | Some present in the group, short to medium duration, | 5 | Enrichment  provided, some use | 5 | Untrained/Some stress or fear noted;  possible  aggression | 5 | Some stress or fear  noted associated with  the training session;  animal did not  participate well | 5 |
| High frequency,  considerable time spend, damage noted | 7 | Non-habituated animal, stress or fear noted ; caught by crush  or box transfer | 7 | Regular presence in the group, short to medium  duration, | 7 | Enrichment  provided, minimal use | 7 | Some stress, fear  and aggression | 7 | Medium levels of stress or fear | 7 |
| High frequency, majority of free time spend,  sizeable damage | 9 | Non-habituated animal, stress or fear evident; caught by crush, net or box transfer | 9 | Often occurrence in the group, medium to long duration | 9 | Enrichment  provided but not used  or adverse reaction  noted | 9 | High levels of  stress and fear  with possible  aggression | 9 | Stress or fear evident | 9 |
| High frequency, majority of free time spend, severe  damage (eg, large wound or digit damage | 10 | Non-habituated animal, stress or fear evident; caught by netting; multiple attempts | 10 | Often occurrence over  more than two days,  medium to long  duration | 10 | No enrichment  provided | 10 | High levels of  stress, fear and  some aggression | 10 | Stress or fear evident. animal  did not participate | 10 |

* Score

**Supplementary Table 3. . Modified Animal Welfare Assessment Grid: Welfare Scoring for environmental Sections**

| Housing | | Group size | | Furnishing/enclosure design | | | Nutrition | | | Access | | Contingent events | | |  |
| --- | --- | --- | --- | --- | --- | --- | --- | --- | --- | --- | --- | --- | --- | --- | --- |
| Good space provision, lighting, ventilation, available shelter, materials  used, temperature, drainage, humidity, UV (if needed) and low noise  levels; enclosure is suitable for the species housed in terms of location, public viewing, proximity to other animals, and so on | S* | Group size in adherence with natural group size;  stocking density  appropriate for the  enclosure; group  structure is suitable | S | | Branches/plants/ hiding places/shelters/nest  boxes and other items  are available allowing  the expression of  natural behaviours | S | | Diet and forage (nutrition)  provided are optimally  suited to the species-specific needs (nutritional, physiological and behavioural) as well as  that of the individual | S | | Access to all of  enclosure | S | Animal movement,  enclosure changes,  building works,  visitor intervention | S | |
| All sutable | 1 | suitable | 1 | | suitable | 1 | | suitable | 1 | | Access to all of  enclosure | 1 | None | 1 | |
| 1-3 factor is below standard | 3 | Group structure differs Slightly, no  overstocking | 3 | | Natural behaviours can be expressed however the options provided for this are minimal | 3 | | The nutrition provided has  reduced suitability to the  individual needs which  would benefit from review | 3 | | Shut in/out good/large  sized enclosure for one day | 3 | External interruption (eg,  ongoing building works, New animal, some change in enclosure furnishing) taking place with minimal  disturbance | 3 | |
| 4-6 factor is below standard | 5 | Group structure differs, Stocking density slightly  higher | 5 | | One type of natural  behaviour is limited | 5 | | The nutrition provided is  suited to the species,  however not the individual | 5 | | Shut in/out  medium-sized  enclosure for part~1 day | 5 | External interruption taking place with some disturbance (Animal moves enclosures, or the enclosure furnishing is taken out and changed) | 5 | |
| 7-8 factor is below standard | 7 | slight  overstocking  Group structure shows a  large difference | 7 | | Numerous natural behaviours is limited | 7 | | The nutrition provided lacks behavioural & physiological requirements | 7 | | Shut in/out enclosure for two to seven days | 7 | External interruption taking place with definite disturbance Multiple new  Animals, | 7 | |
| 9-10 factor is below standard | 9 | Group structure shows a large difference with Stocking density higher | 9 | | Natural behaviours are very limited | 9 | | The nutrition provided lacks nutritional requirements of the species | 9 | | Shut into small indoor/  outdoor enclosure for part~1 day | 9 | External interruption taking place with definite  disturbance over more than seven days | 9 | |
| All factors scored  inadequate | 10 | Large difference  (Solitary housing  of social animals) | 10 | | The options are not  provided | 10 | | The nutrition provided is not suited to the requirements of the species and the individua | 10 | | Shut into small indoor/ outdoor enclosure for  more than one day or medium/large  enclosure for more than seven days | 10 | Animal introduced into new enclosure and group, with ongoing external disturbance | 10 | |

* Score

**Supplementary Table 4. Modified Animal Welfare Assessment Grid: Welfare Scoring for procedural Sections**

| Restraint | | Sedation | | Time bird  restrained before/  during procedure | | | Veterinary procedure | | | Change in daily routine | | | Visitor score––  number, noise  level, adverse  interactions | | |  |
| --- | --- | --- | --- | --- | --- | --- | --- | --- | --- | --- | --- | --- | --- | --- | --- | --- |
| Train/ habituated  to restraint | S* | Sedation stress,  recovery | S | | Time bird restrained before/ during procedure | S | | Degree of veterinary procedure, effect | S | | Starving hours, changing housing, solitary housing | S | | Visitor number, noise level, adverse interaction | S | |
| Trained animal, no stress  or fear noted; positive  reinforcement used | 1 | No sedation | 1 | | Procedure  performed  immediately while  bird in hand after  catching | 1 | | No veterinary procedure | 1 | | No change | 1 | | No visitors | 1 | |
| Trained animal, some  stress or fear noted | 3 | Calm induction and recovery and  rapid return to normal | 3 | | Bird held in crate  <30 minutes  before procedure | 3 | | Minor veterinary procedure  with noticeable short-term  effect on animal | 3 | | Starved for <6 hours, Change in housing for the aftermath of the procedure (max 4 hours) | 3 | | <50 visitors during hr obs moderately increased noise  level | 3 | |
| Non-habituated animal;  no stress / Trained/habituated  animal; some stress or  fear noted | 5 | Stressful induction and/or recovery but  rapid return to normal for a few hours  after event | 5 | | Bird held in crate  30–60 minutes  before procedure | 5 | | Moderate veterinary procedure with noticeable short-term or medium-term effect on anima | 5 | | Change in housing for the aftermath of the procedure (max 12 hours) | 5 | | 50–100 visitors  during hr obs little  increased noise  level | 5 | |
| Non-habituated animal;  some stress or fear noted/ | 7 | Sedation with either stressful induction/recovery and/or effects on normal behaviour and medium-term effects on  integration with the group afterwards | 7 | | Bird held in crate  >1 hour before  procedur | 7 | | Moderate veterinary procedure with noticeable long-term effect on animal including loss of group status | 7 | | Starved between 6–12 hours in primates; change in housing for the aftermath of the procedure (max 12 hours) | 7 | | 50–100 visitors  during hr obs greatly increased noise level or adverse  interactions | 7 | |
| Trained/habituated animal; stress or fear evident | 9 | Prolonged sedation with highly stressful induction and/ or recovery and moderate  long-term effects on normal behaviour and affecting integration with the group | 9 | | Bird held in crate  1–2 hours before  procedure | 9 | | Extensive veterinary procedure resulting in severe short-term pain despite appropriate treatment and analgesia  and a loss of status in the  hierarchy | 9 | | Change in housing  (>12 hours) (solitary in birds) and starved >12 hours (in primates) | 9 | | >100 visitors during hr obs moderately  increased noise  level | 9 | |
| Non-habituated animal;  stress or fear evident; | 10 | Prolonged sedation with highly stressful induction and/ or recovery and prolonged  effects on normal behaviour and food intake afterwards and/or preventing integration  with the group | 10 | | Bird held in crate  >2 hours before  procedure | 10 | | Extensive veterinary procedure resulting in severe long-term pain despite appropriate treatment and analgesia  and a loss of status in the  hierarch | 10 | | Change in housing  (>12 hours) (>24 hours in birds), starved for >12 hours (in primates),  solitary housing | 10 | | >100 visitors during hr obs greatly increased noise level or adverse interactions | 10 | |

* Score

Supplementary Table 5. Results of ANOVA about the statistical difference of four AWAG section by species across the four level of zoos (A, B, C & D) in South Korea (α = 0.05).

|  |  | A | B | C | D | *P* | Post hoc |
| --- | --- | --- | --- | --- | --- | --- | --- |
| Japanse macaques | Physical  Psychological  Procedure  Environmental | 1.80±0.42  3.35±0.49  4.80±0.71  4.60±0.28 | 2.98±1.59  4.53±0.93  5.38±1.22  4.98±0.42 | 2.15±0.66  4.60±1.57  6.78±1.65  4.35±0.90 | 2.25±1.06  5.35±1.36  7.80±0.81  3.58±0.90 | 1. 0.861   2. 0.344  3. 0.089  4. 0.468 | 1.A=B=C=D  2.A=B=C=D  3.A=B=C=D  4.A=B=C=D |
| Tigers | Physical  Psychological  Procedure  Environmental | 1.00±0.00  2.50±1.13  4.60±1.41  4.20±0.71 | 1.73±0.79  3.73±0.50  5.35±0.55  4.70±0.45 | 2.25±0.64  4.20±0.71  4.80±0.00  5.75±2.48 | None | 1. 0.183  2. 0.088  3. 0.607  4. 0.388 | 1.A=B=C  2.A=B=C  3.A=B=C  4.A=B=C |
| Prairie dogs | Physical  Psychological  Procedure  Environmental | 1.30±0.28  1.95±0.50  4.00±0.00  2.65±0.49 | 1.80±0.57  3.53±1.21  3.80±0.28  4.30±2.26 | 2.70±0.46  3.53±1.21  4.03±0.59  7.37±0.35 | 2.76±1.23  4.30±0.71  3.84±1.17  8.42±1.28 | 1. 0.109  2. 0.044^*^  3. 0.723  4. 0.040^*^ | 1. A=B=C=D  2. D>A, D>B  3. A=B=C=D  4. D>A, D>B |
| Meerkats | Physical  Psychological  Procedure  Environmental | 1.05±0.70  2.05±0.49  3.60±0.00  3.15±0.21 | 1.28±0.25  3.18±0.67  4.13±0.19  4.58±2.02 | 2.00±0.75  3.18±0.59  4.45±0.25  7.20±0.78 | 3.10±1.00  4.43±0.78  4.03±0.37  8.45±0.64 | 1. 0.008  2. 0.010^*^  3. 0.045^*^  4. 0.009^*^ | 1. D>A, D>B   2. D>A  3. C>A  4. D>A, D>B |
| Fennec foxes | Physical  Psychological  Procedure  Environmental | 1.45±0.70  3.00±0.00  4.60±0.28  4.00±0.42 | 2.38±1.20  4.53±1.30  4.93±0.64  5.98±1.97 | 2.13±0.64  3.73±0.64  4.30±0.20  7.80±0.14 | 4.07±1.85  5.20±1.32  3.87±0.50  8.27±0.83 | 1. 0.202  2. 0.112  3. 0.092  4. 0.121 | 1.A=B=C=D  2.A=B=C=D  3.A=B=C=D  4.A=B=C=D |
| Rabbit | Physical  Psychological  Procedure  Environmental | 1.10±0.14  3.00±0.42  3.60±0.00  5.00±0.99 | 1.73±0.46  2.98±0.59  3.63±0.37  5.00±0.85 | 1.22±0.39  3.45±0.45  3.88±0.34  6.78±0.75 | 2.48±0.64  3.70±0.40  3.03±0.60  8.07±0.55 | 1. 0.021^*^  2. 0.128  3. 0.140  4. 0.007^*^ | 1.D>C  2.A=B=C=D  3. A=B=C=D  4. D>B |
| Leopard tortoise | Physical  Psychological  Procedure  Environmental | 1.10±0.00  3.15±0.21  3.30±0.42  4.70±0.00 | 2.73±0.76  3.33±0.91  4.10±0.17  6.50±1.05 | 2.85±1.97  3.95±0.54  4.13±0.25  7.13±0.43 | 2.64±0.80  3.66±0.35  3.22±0.79  7.54±0.53 | 1. 0.172  2. 0.257  3. 0.035^*^  4. 0.790 | 1.A=B=C=D  2.A=B=C=D  3.A=B=C=D  4.A=B=C=D |
| Macaw | Physical  Psychological  Procedure  Environmental | 1.35±0.35  2.80±0.57  3.75±0.35  4.20±0.71 | 3.20±1.52  5.03±0.42  4.23±0.53  5.70±1.21 | 1.47±0.47  2.93±0.55  3.60±0.17  7.17±1.68 | 4.00±1.22  5.43±0.38  3.50±0.92  9.10±0.66 | 1. 0.071  2. 0.036^*^  3. 0.348  4. 0.055 | 1.A=B=C=D  2.A=B=C=D  3.A=B=C=D  4.A=B=C=D |
| Raccoon quati | Physical  Psychological  Procedure  Environmental | 1.15±0.07  2.75±0.70  4.60±0.28  3.05±0.70 | 1.68±0.25  3.08±0.78  4.43±0.33  4.83±0.42 | 1.60±0.52  3.78±0.88  4.68±0.49  6.78±1.37 | 2.78±1.62  4.46±1.62  3.66±0.49  7.68±0.86 | 1. 0.362  2. 0.273  3. 0.040^*^  4. 0.014 | 1.A=B=C=D  2.A=B=C=D  3.A=B=C=D  4.A=B=C=D |
| Corn snake | Physical  Psychological  Procedure  Environmental | 1.10±0.14  1.00±0.00  4.05±0.49  3.25±0.07 | 1.70±0.42  3.35±0.49  4.80±0.57  5.05±0.07 | 1.67±0.83  3.43±0.23  4.13±0.23  5.90±0.85 | 1.82±0.75  3.54±0.45  3.60±0.49  6.30±0.94 | 1. 0.353  2. 0.176  3. 0.116  4. 0.050 | 1.A=B=C=D  2.A=B=C=D  3.A=B=C=D  4.A=B=C=D |
| Cockatiel | Physical  Psychological  Procedure  Environmental | 1.00±0.00  1.80±0.71  3.40±0.28  3.15±0.21 | 1.47±0.45  3.43±0.81  3.40±0.35  4.57±1.42 | 3.60±0.00  3.47±0.31  3.47±0.23  8.17±1.67 | 3.28±2.14  4.94±2.08  3.38±1.06  8.88±0.61 | 1. 0.041^*^  2. 0.128  3. 0.994  4. 0.028^*^ | 1.A=B=C=D  2.A=B=C=D  3.A=B=C=D  4.A=B=C=D |
